# Active and passive maternal smoking during pregnancy and birth outcomes: the Kyushu Okinawa Maternal and Child Health Study

**DOI:** 10.1186/1471-2393-13-157

**Published:** 2013-08-06

**Authors:** Yoshihiro Miyake, Keiko Tanaka, Masashi Arakawa

**Affiliations:** 1 Department of Preventive Medicine and Public Health, Faculty of Medicine, Fukuoka University, Fukuoka, Japan; 2 Course of Wellness, Graduate School of Tourism Sciences, University of the Ryukyus, Okinawa, Japan

## Abstract

**Background:**

In Western countries, active maternal smoking during pregnancy is recognized as the most important preventable risk factor for adverse birth outcomes. However, the effect of passive maternal smoking is less clear and has not been extensively studied. In Japan, there has been only one epidemiological study which examined the effects of active smoking during early pregnancy on birth outcomes although the effects of passive smoking were not assessed.

**Methods:**

Study subjects were 1565 mothers with singleton pregnancies and the babies born from these pregnancies. Data on active maternal smoking status in the first, second, and third trimesters and maternal environmental tobacco smoke (ETS) exposure at home and work were collected with self-administered questionnaires.

**Results:**

Compared with children born to mothers who had never smoked during pregnancy, children born to mothers who had smoked throughout their pregnancy had a significantly increased risk of small-for-gestational-age (SGA) (adjusted odd ratio [OR] = 2.87; 95% confidence interval: 1.11 − 6.56). However, active maternal smoking only in the first trimester and active maternal smoking in the second and/or third trimesters but not throughout pregnancy were not significantly associated with SGA. With regard to the risk of preterm birth, the adjusted ORs for the above-mentioned three categories were not significant; however, the positive linear trend was significant (*P* for trend = 0.048). No significant association was found between active maternal smoking during pregnancy and the risk of low birth weight. There was a significant inverse relationship between active maternal smoking during pregnancy and birth weight; newborns of mothers who had smoked throughout pregnancy had an adjusted mean birth weight reduction of 169.6 g. When classifying babies by gender, a significant positive association between active maternal smoking throughout pregnancy and the risk of SGA was found only in male newborns, however, the interaction was not significant. Maternal ETS exposure at home or work was not significantly associated with any birth outcomes.

**Conclusions:**

This is the first study in Japan to show that active maternal smoking throughout pregnancy, but not during the first trimester, is significantly associated with an increased risk of SGA and a decrease in birth weight. Thus, women who smoke should quit smoking as soon as possible after conception.

## Background

Growth-restricted infants are at increased risk for perinatal mortality and morbidity, short stature, cognitive delays, and neurologic disorders [1]. A meta-analysis showed significant inverse associations between birth weight and adult mortality from all-causes and cardiovascular mortality [2]. In Japan, low birth weight (LBW) is an important public health issue; the LBW rate declined until the 1970s, reaching 5.2% in 1978–1979, but then increased again to reach 9.5% in 2005 [3]. This fact prompted us to examine risk factors for outcomes in relation to birth weight.

Many studies conducted in Western countries have found a significant inverse relationship between active maternal smoking during pregnancy and birth weight and/or significant positive associations between active maternal smoking during pregnancy and the risk of LBW, preterm birth, and/or small-for-gestational-age (SGA) [4-24]. To the best of our knowledge, only one study in Japan has examined the association between smoking during early pregnancy and birth outcomes [25]. In several studies in the West, quitting maternal smoking in early pregnancy was found not to affect the risk of LBW, preterm birth, and/or SGA [5,6,8,22-24]. On the other hand, the effect of passive maternal smoking during pregnancy is less clear and has not been extensively studied [8,12,16,18,22,26]. In a meta-analysis performed in 2010, a significant association was found between environmental tobacco smoke (ETS) exposure and lower mean birth weight (weighted mean difference: −60 g; 95% confidence interval [CI]: −89 to −39 g) although ETS exposure was not significantly related to the risk of LBW or preterm delivery [27].

The current prebirth cohort study was performed to address the paucity of epidemiological information regarding the relationship between active and passive maternal smoking during pregnancy and birth outcomes in Japan using data from the Kyushu Okinawa Maternal and Child Health Study (KOMCHS).

## Methods

### Study population

The KOMCHS was a prospective prebirth cohort study performed to examine risk and preventive factors for maternal and child health problems. Eligible women were those who became pregnant and lived in seven prefectures on Kyushu Island in southern Japan and Okinawa Prefecture. Between April 2007 and March 2008, we requested that 131 obstetric hospitals in Fukuoka Prefecture, the largest prefecture on Kyushu Island with a total population of approximately 5.04 million, provide as many pregnant women as possible with a set of leaflets explaining the KOMCHS, an application form to participate in the study, and a self-addressed and stamped return envelope. Between May 2007 and March 2008, we also requested that 40 obstetric hospitals in Okinawa Prefecture, an island chain in the southwest of Japan with a total population of nearly 1.37 million, provide as many pregnant women as possible with the same documents. Between August 2007 and March 2008, pregnant women living in six prefectures on Kyushu Island other than Fukuoka Prefecture, with a total population of approximately 8.22 million, were also provided with the same documents at 252 obstetric hospitals. Pregnant women who were willing to participate in the study returned the application form containing a written description of their personal information to the data management center. Based on the personal information, research technicians gave them a detailed explanation of the KOMCHS by telephone and sent them a set of two self-administered questionnaires after obtaining their agreement. In total, 1757 pregnant women between the 5th and 39th weeks of pregnancy gave their full informed consent in writing to participate in the KOMCHS and completed the baseline survey. Of the 1757 women, 1590 mother-child pairs took part in the second survey, which was carried out after delivery: 73.5% of the mothers completed the second survey at less than 1 month of giving birth, while 12.9%, 7.4%, 3.3%, and 2.9% completed the second survey at 1, 2, 3, and 4–11 months of giving birth, respectively. Excluded from the current analysis were 23 mothers with multiple births and 2 mothers in which the gender of the baby was not known due to incomplete data reporting. The final study group thus consisted of 1565 mother-child pairs. The ethics committee of the Faculty of Medicine, Fukuoka University approved the KOMCHS.

### Measurements

Two questionnaires were administered to each participating mother. The first questionnaire, which consisted of two parts, was filled out by mothers during pregnancy (between the 5th and 39th week of pregnancy). The second questionnaire was filled out by mothers after giving birth. Participants mailed these questionnaires to the data management center conducting each survey. Research technicians clarified or completed missing or unclear data by telephone.

The first questionnaire, completed during pregnancy, elicited information about maternal age; region of residence; number of children; family structure; maternal education; maternal employment status; and maternal ETS exposure at home and at work. Employment status in the year when the first questionnaire was conducted or in the previous year was also elicited and women were classified as being unemployed if they were unemployed both in the year the questionnaire was completed and the preceding year. The second part of the first questionnaire was a semi-quantitative, comprehensive dietary history questionnaire that assessed dietary habits during the month preceding the time when the mother completed the questionnaire. Body weight and height were self-reported in this questionnaire. Body mass index was calculated as weight (kg) divided by the square of height (m).

In Japan, generally, an obstetrician’s estimate of gestational age at the time of delivery is based on early ultrasound examination and/or the first day of the last menstrual period and birth weight is measured right after birth. Data such as birth weight and gestational age at birth are recorded by staff at the birth hospital or clinic in a booklet called the Maternal and Child Health Handbook. These booklets are provided to every pregnant woman by the municipality in which the woman resides when the women become pregnant and they contain data pertaining to prenatal checkups, postnatal health condition of both mother and baby, and growth of the child.

The second self-administered questionnaire elicited information on active maternal smoking status in the first (≤ 15 weeks’ gestation), second (16–27 weeks’ gestation), and third (≥ 28 weeks’ gestation) trimesters; gestational age; birth weight; and baby’s gender. With regard to gestational age and birth weight, mothers were advised to refer to their Maternal and Child Health Handbook.

With regard to outcomes, low birth weight was defined as a birth weight less than 2500 g. Preterm birth was defined as a birth occurring at a gestational age of less than 37 weeks. SGA was defined as a birth weight below the 10th percentile of the Japanese neonatal anthropometric norms for babies of the same gestational age, gender, and parity published by Itabashi et al. in 2010 [28]. These norms show the distribution of birth weights for each day of gestational age; however, data on gestational ages were only available by weeks in this study. Thus, for the purposes of comparison and analysis, we selected the distributions from the third day of each week from the study of Itabashi et al.

### Statistical analysis

Maternal age at the time of the first questionnaire; region of residence; number of children; family structure; maternal education; maternal employment status; maternal alcohol consumption during the preceding month; body mass index; gestational age at birth; and baby’s gender were selected as *a priori* potential confounding factors; however, gestational age at birth was removed when the associations with the risk of preterm birth were examined. Marital status was not taken into consideration because 96.3% of the mothers lived with their husbands and there was only one single mother in the study. Active maternal smoking was classified into four categories: 1) never smoked during pregnancy, 2) smoked only in the first trimester, 3) smoked in the second and/or third trimesters regardless of smoking status in the first trimester but not throughout the pregnancy, and 4) smoked throughout pregnancy. Maternal ETS exposure at home was classified into two categories: 1) no one smoked in the mother’s home at the time of the first questionnaire, and 2) someone smoked in the mother’s home at the time of the first questionnaire. Maternal ETS exposure at work was classified into two categories: 1) no one smoked in the mother’s workplace at the time of the first questionnaire, and 2) at least one person smoked in the mother’s workplace at the time of the first questionnaire. Region of residence was classified into three categories: 1) Fukuoka Prefecture, 2) prefectures other than Fukuoka Prefecture on Kyushu Island, and 3) Okinawa Prefecture. Number of living children previously born to the same mother (ie, siblings) was classified into three categories: 0, 1 or 2 or more. Family structure was classified into nuclear or extended. Maternal education was classified into three categories: 1) < 13 years, 2) 13–14 years, and 3) ≥ 15 years. Maternal employment was classified as yes or no. And, finally, alcohol consumption during the month preceding the filling out of the first questionnaire was classified as yes or no. Maternal age, body mass index, and gestational age at birth were used as continuous variables.

Multiple logistic regression analysis was used to estimate adjusted odds ratios (ORs) and 95% CIs for birth outcomes according to active and passive maternal smoking status. Analysis of covariance was used to calculate adjusted means of birth weights according to active and passive maternal smoking status with allowance for confounding factors. Trends of an association were assessed with a multiple logistic regression model or a multiple linear regression analysis assigning consecutive integers to the categories of the exposure variables. All statistical analyses were performed using the SAS software package version 9.2 (SAS Institute, Inc., Cary, NC, USA).

The analysis of the effects of ETS exposure at home and at work on birth outcomes was based on the 1427 mother-child pairs who had never smoked during pregnancy.

## Results

Maternal mean age at the time of the first questionnaire was 31.3 years (Table 1). The mean birth weight of the babies, as reported in the second questionnaire, was 3006.3 g. Among the 1565 newborns, 120 (7.7%) were classified as LBW, 62 (4.0%) as preterm birth, and 122 (7.8%) as SGA.

**Table 1:** Distribution of selected characteristics in 1565 mother-child pairs

**Variable**	***n *(%)**
Baseline characteristics	
Maternal age, years, mean ±SD	31.3 ± 4.2
Region of residence	
Fukuoka Prefecture	883 (56.4)
Prefecture on Kyushu Island other than Fukuoka	527 (33.7)
Okinawa Prefecture	155 (9.9)
Number of living children already born to same mother	
0	615 (39.3)
1	633 (40.5)
≥ 2	317 (20.3)
Nuclear family structure	1336 (85.4)
Maternal education, years	
< 13	361 (23.1)
13 − 14	523 (33.4)
≥ 15	681 (43.5)
Maternal employment^a^	943 (60.3)
Alcohol consumption during the preceding month	209 (13.4)
Body mass index, kg/m^2^, mean ± SD	21.4 ± 2.7
Infant characteristics at birth	
Birth weight, g, mean ± SD	3006.3 ± 395.5
Gestational age, weeks, mean ± SD	38.9 ± 1.5
Male gender	762 (48.7)
Low birth weight (< 2500 g)	120 (7.7)
Preterm birth (< 37 weeks)	62 (4.0)
Small-for-gestational-age (< 10th percentile)	122 (7.8)

^a^ Full-time or part-time employment in the year when the first questionnaire was conducted or in the previous year.

Table 2 provides adjusted ORs for LBW, preterm birth, and SGA and adjusted means of birth weight according to active maternal smoking status during pregnancy. The median number of cigarettes smoked per day in the first, second, and third trimester was, respectively, 10, 5, and 5 cigarettes. Compared with a reference group of mothers who had never smoked during pregnancy, those who had smoked throughout their pregnancy had a significantly increased risk of SGA children (adjusted OR = 2.87; 95% CI: 1.11 − 6.56) while those who smoked only in the first trimester had a non-significantly reduced risk of SGA. Women who smoked in the second and/or third trimesters but who did not smoke throughout their pregnancy had a 1.9-fold increased risk of SGA (but this did not reach statistical significance). But, the positive linear trend was significant (*P* for linear trend = 0.04). An exposure-response relationship between active maternal smoking and the risk of preterm birth was significant (*P* for linear trend = 0.048) although the adjusted ORs for the above-mentioned three categories were not significant. No significant association was found between active maternal smoking and LBW. There was a significant inverse relationship between active maternal smoking and birth weight: newborns of mothers who had smoked throughout their pregnancy had adjusted mean birth weight reductions of 169.6 g (*P* for trend = 0.005).

**Table 2:** Adjusted odds ratios (ORs) and 95% confidence intervals (CIs) for birth outcomes and adjusted means of birth weight according to active maternal smoking status during pregnancy in 1565 mother-child pairs

	**Low birth weight**		**Preterm birth**		**Small-for-gestational-age**		**Adjusted mean of birth weight, g (95% CI)**^**a**^
**Maternal smoking**	**Rate (%)**	**OR (95% CI)**^**a**^	**Rate (%)**	**OR (95% CI)**^**b**^	**Rate (%)**	**OR (95% CI)**^**a**^	
None (n = 1427)	7.4	1.00	3.5	1.00	7.6	1.00	3010.7 (2994.0 − 3027.4)
First trimester only (n = 71)	7.0	0.52 (0.12 − 1.65)	8.5	2.51 (0.90 − 5.98)	4.2	0.53 (0.13 − 1.49)	3027.9 (2951.4 − 3104.3)
Second and/or third trimesters but not throughout (n = 28)	21.4	2.75 (0.71 − 8.89)	10.7	3.14 (0.71 − 9.80)	14.3	1.93 (0.55 − 5.27)	2958.3 (2837.9 − 3078.7)
Throughout (n = 39)	10.3	2.17 (0.48 − 7.14)	7.7	2.06 (0.47 − 6.34)	18.0	2.87 (1.11 − 6.56)	2841.1 (2738.4 − 2943.8)
*P* for trend		0.19		0.048		0.04	0.005

^a^ Adjustment for maternal age; region of residence; number of children; family structure; maternal education; maternal employment; alcohol consumption during the preceding month; body mass index; gestational age; and baby’s gender.

^b^ Adjustment for maternal age; region of residence; number of children; family structure; maternal education; maternal employment; alcohol consumption during the preceding month; body mass index; and baby’s gender.

When classifying babies by gender, a significant positive association between active maternal smoking throughout pregnancy and the risk of SGA was found only in male newborns, however, the interaction between active maternal smoking throughout pregnancy and infant gender with respect to SGA was not statistically significant (*P* for interaction = 0.34; Table 3). Also, a significant inverse relationship between active maternal smoking and birth weight was observed only in male newborns; however, the decrease in adjusted mean birth weight among babies of mothers who had smoked throughout pregnancy was greater in girls than in boys (−182.4 g vs. −149.2 g, respectively).

**Table 3:** Adjusted odds ratios (ORs) and 95% confidence intervals (CIs) for small-for-gestational-age and adjusted means of birth weight according to active maternal smoking status during pregnancy by baby’s gender in 1565 mother-child pairs

	**Male (n = 762)**			**Female (n = 803)**		
**Maternal smoking**	**Rate (%)**	**OR (95% CI)**^**a**^	**Adjusted mean of birth weight, g (95% CI)**^**a**^	**Rate (%)**	**OR (95% CI)**^**a**^	**Adjusted mean of birth weight, g (95% CI)**^**a**^
None	7.1 (n = 703)	1.00	3058.8 (3034.3 − 3083.3)	8.0 (n = 724)	1.00	2964.3 (2941.4 − 2987.3)
First trimester only	7.1 (n = 28)	1.02 (0.16 − 3.81)	3054.9 (2930.2 − 3179.5)	2.3 (n = 43)	0.24 (0.01 − 1.22)	3004.2 (2906.8 − 3101.5)
Second and/or third trimesters but not throughout	12.5 (n = 8)	1.67 (0.08 − 11.08)	2981.7 (2748.3 − 3215.0)	15.0 (n = 20)	2.14 (0.48 − 6.92)	2919.5 (2780.2 − 3058.9)
Throughout	21.7 (n = 23)	4.21 (1.26 − 12.14)	2909.6 (2771.0 − 3048.2)	12.5 (n = 16)	1.51 (0.23 − 5.96)	2781.9 (2625.9 − 2937.9)
*P* for trend		0.03	0.04		0.61	0.09

^a^ Adjustment for maternal age; region of residence; number of children; family structure; maternal education; maternal employment; alcohol consumption during the preceding month; body mass index; and gestational age.

Results for maternal ETS exposure at the time of the first questionnaire in the sample of 1427 mother-child pairs in which the mother had never smoked during pregnancy are given in Table 4. Maternal ETS exposure at home or at work was not significantly associated with the risk of any adverse birth outcomes. Also, there was no relationship found between maternal ETS exposure at home or at work and birth weight.

**Table 4:** Adjusted odds ratios (ORs) and 95% confidence intervals (CIs) for birth outcomes and adjusted means of birth weight according to maternal environmental tobacco smoke (ETS) exposure at the time of the first questionnaire in 1427 mother-child pairs for mothers who never smoked during pregnancy

**Passive maternal smoking status**	**Low birth weight**		**Preterm birth**		**Small-for-gestational-age**		**Adjusted mean of birth weight, g (95% CI)**^**a**^
	**Rate (%)**	**OR (95% CI)**^**a**^	**Rate (%)**	**OR (95% CI)**^**b**^	**Rate (%)**	**OR (95% CI)**^**a**^	
ETS exposure at home							
No (n = 911)	7.4	1.00	3.5	1.00	8.1	1.00	3014.3 (2993.0 − 3035.6)
Yes (n = 516)	7.4	0.88 (0.53 − 1.42)	3.5	0.91 (0.48 − 1.65)	6.6	0.80 (0.51 − 1.24)	3011.2 (2982.6 − 3039.7)
*P*^c^							0.86
ETS exposure at work							
No (n = 1255)	7.5	1.00	3.5	1.00	7.7	1.00	3010.4 (2992.4 − 3028.4)
Yes (n = 172)	6.4	0.97 (0.44 − 1.94)	3.5	0.97 (0.36 − 2.23)	7.0	0.96 (0.48 − 1.78)	3033.2 (2983.1 − 3083.2)
*P*^c^							0.41

^a^ Adjustment for maternal age; region of residence; number of children; family structure; maternal education; maternal employment; alcohol consumption during the preceding month; body mass index; gestational age; and baby’s gender.

^b^ Adjustment for maternal age; region of residence; number of children; family structure; maternal education; maternal employment; alcohol consumption during the preceding month; body mass index; and baby’s gender.

^c^*P* value was calculated by analysis of covariance.

## Discussion

The current study demonstrated that active maternal smoking throughout pregnancy poses a statistically significant increased risk for SGA, especially in male newborns. However, in this study, we found that active maternal smoking only in the first trimester, or active maternal smoking in the second and/or third trimesters did not pose a statistically significant risk for SGA. The abovementioned three categories in relation to active maternal smoking were not significantly related to the risk of preterm birth, although the positive linear trend was statistically significant. There was no significant relationship between active maternal smoking and LBW. Active maternal smoking throughout pregnancy was significantly associated with a decrease in birth weight, with an adjusted mean reduction in birth weight of 169.6 g. No significant associations were found between maternal ETS exposure at home or work and any of the birth outcomes we studied.

A prebirth cohort study in Greece showed that active maternal smoking at 12 weeks of gestation was significantly positively related to the risk of LBW and SGA, but not preterm birth, while smoking cessation before 12 weeks of gestation was not associated with the risk of preterm birth, LBW, or SGA [5]. In a prebirth cohort study conducted in New Zealand and Australia, women who were still smoking at 15 weeks of gestation had a significantly increased risk for preterm birth and SGA compared with those who had stopped smoking before 15 weeks gestation [6]. The same study found that for those who stopped smoking before 15 weeks of gestation had the same risk of preterm birth and SGA as women who did not smoke at all during pregnancy. Continued active smoking after conception is known to be significantly positively associated with the risk of preterm birth and LBW, while smoking until conception was found not to be related to the risk of preterm birth or LBW in the Generation R Study conducted in The Netherlands [8]. In a historical cohort study in Brazil, active maternal smoking during the whole pregnancy was significantly associated with an increased risk of LBW and SGA, but not preterm birth, while smoking cessation during the first trimester was found to have no statistically significant relationship with increased risk of preterm birth, LBW, or SGA [22]. A prebirth cohort study conducted in the USA found that women who smoked throughout their pregnancy had a significantly increased risk of SGA, while the risk of SGA for those who had smoked only during the first trimester was similar to that of non-smokers [23]. In a retrospective cohort analysis of US birth certificates, which compared women who smoked throughout pregnancy with those who quit during their pregnancy, those who quit smoking in the first trimester had lower risk of delivering preterm non-SGA, term SGA, and preterm SGA newborns, and this risk was similar in magnitude to those who never smoked [24]. The present results concerning active maternal smoking during pregnancy are in partial agreement with these observations. In particular, the current results confirm prior research that has demonstrated that those who quit smoking in early pregnancy can achieve the same low risk of adverse birth outcomes as those who never smoke during pregnancy.

The difference we found in the effect of maternal smoking on male and female babies differs from the findings of a study in Germany that found that, particular in heavy smokers, the adverse effect of maternal smoking during pregnancy on the mean birth weight and risk of SGA was greater in girls than in boys [10].

With respect to maternal ETS exposure during pregnancy among mothers who never smoked during pregnancy, the current results are consistent with those of some previous studies that showed no relationship between passive maternal smoking during pregnancy and birth outcomes [12,18,26]. In the Generation R Study, however, maternal ETS exposure of more than 3 hours per day in late pregnancy (≥ 25 weeks’ gestation) was significantly associated with an increased risk of LBW, but not preterm birth [8]. A case–control study in New Zealand showed a significant positive relationship between the risk of SGA and maternal ETS exposure in the workplace or while socializing, but not between the risk of SGA and paternal or other household smoking [16]. Women whose partner smoked during pregnancy had a significantly increased risk of SGA, but not LBW or preterm birth in the previously cited Brazilian study [22]. Our results are in partial agreement with these findings. The discrepancies among studies may be explained, at least in part, by differences in characteristics, smoking habits, and lifestyle of the populations examined.

According to a review by Jauniaux and Burton, tobacco toxins interfere with the trophoblastic and biological functions of fetal cells that regulate protein metabolism and enzyme activity, leading to an impact on fetal growth, with a reduction of weight, body fat, and many other anthropometric parameters [29]. We have no ready explanation as to why the detrimental effect of active maternal smoking on the risk of SGA was more evident in male babies than in female babies in the present study. The greater male susceptibility might be explained by a different hormonal milieu.

Several weaknesses of the present study warrant mention. Information on tobacco exposure was obtained with self-administered questionnaires and was not validated by objective measurements such as cotinine levels. However, Pickett et al. reported that urinary cotinine measures and self-reported number of cigarettes were highly correlated at any given time point in pregnant women in the USA [30]. Although data on maternal smoking status at different trimesters were available, such data were collected using the second questionnaire after birth. Consequently, the possibility of recall bias should be considered; however, any resulting exposure misclassification would be non-differential and would have yielded an underestimation of values in our results.

In the first questionnaire, we could not estimate the participation rate because we do not have exact figures for the number of pregnant women who were provided with a set of leaflets explaining the KOMCHS, an application form, and a self-addressed and stamped return envelope by the 423 collaborating obstetric hospitals. This situation made it impossible to assess the differences between participants and non-participants, because no information on personal characteristics such as maternal age and socioeconomic status was available for non-participants. Our subjects were probably not representative of Japanese women in the general population. For example, a population census conducted in 2000 in Fukuoka Prefecture found that the percentages of women aged 30 to 34 years with < 13, 13–14, ≥ 15, and an unknown number of years of education were 52.0%, 31.5%, 11.8%, and 4.8%, respectively [31]. The corresponding figures for this study were 23.1%, 33.4%, 43.5%, and 0.0%, respectively. Thus, our study subjects were more educated and probably more aware of health topics than women in the general population. Nevertheless, cigarette-smoking status in our study population was likely to be similar to that of the general population. In the National Health and Nutrition Survey in Japan of 2007, the percentages of currently-smoking, formerly-smoking, and non-smoking women aged 30 to 39 years were 17.2%, 11.4%, and 71.4%, respectively, although data specific to pregnant women were not available [32]. At baseline, 31.7% of the present study subjects had ever smoked.

The current study did not have substantial statistical power although significant associations were detected. Although adjustment was made for several confounding factors, residual confounding effects could not be ruled out.

## Conclusions

To the best of our knowledge, this is the first study in Japan to show that active maternal smoking throughout pregnancy is significantly associated with an increased risk of SGA and a decrease in birth weight while maternal smoking only in the first trimester was not related to these outcomes. Taking the results of the present study together with those of previous epidemiological studies, women who smoke should quit smoking as soon as possible after conception.

## Abbreviations

CI: Confidence interval; ETS: Environmental tobacco smoke; KOMCHS: Kyushu Okinawa Maternal and Child Health Study; LBW: Low birth weight; OR: Odds ratio; SGA: Small-for-gestational-age.

## Competing interests

All authors declare that they have no competing interests.

## Authors’ contributions

YM, KT, and MA contributed to the study concept and design and the acquisition of data. YM was responsible for the analysis and interpretation of data and the drafting of the manuscript. All authors participated in critically revising the manuscript and approved the final version of the manuscript.

## Pre-publication history

The pre-publication history for this paper can be accessed here:

http://www.biomedcentral.com/1471-2393/13/157/prepub
